# Impact of COVID-19 on gynaecological patient care: results of patient’s survey with 327 patients

**DOI:** 10.1007/s00404-021-06280-8

**Published:** 2021-10-27

**Authors:** F. Recker, S. Dohmen, E. K. Egger, M. B. Stope, D. Dimitrova, D. Könsgen, M. Ritter, J. Sehouli, M. Gadebusch Bondio, A. Mustea

**Affiliations:** 1 Department for Gynecology and Gynecological Oncology, University Hospital Bonn, Venusberg Campus 1, 53127 Bonn, Germany; 2 Department of Urology, University Hospital Bonn, Bonn, Germany; 3 Department for Gynecology with Centre for Oncological Surgery, Campus Virchow Klinikum, Berlin, Germany; 4 Institute of Medical Humanities, University Hospital Bonn, Bonn, Germany

**Keywords:** COVID-19, Patients’ care, Gynaecology

## Abstract

**Purpose:**

The pandemic SARS-CoV-2 poses new and unprecedented challenges for health care systems on a national and global level. Although the current situation has been going on for more than 1 year, there is limited data on the impact of the pandemic on general hospital and medical practice care. This survey captures the perspective of patients with gynaecological diseases of this impact.

**Methods:**

Using a paper-based questionnaire, 327 patients were asked about medical care and their experiences during the pandemic at the University Hospital Bonn and the University Hospital Charité Berlin. The study was performed from the 1st June to 30th September 2020.

**Results:**

A total of 327 patients participated in the study: 156 stated to have been tested for coronavirus, and 1 patient reported a positive test. 41.3% of the patients felt insecure about the current situation, 30.4% were concerned about the risk of infection during the hospital stay. The pandemic-specific measures in hospitals and medical practices unsettled 6.8% of patients. 18.1% of patients feared that their gynaecological disease would not be treated adequately due to the pandemic. 55.7% of patients reported that their confidence in their physicians has increased during the pandemic.

**Conclusion:**

The results show that patients’ confidence in the healthcare system and the physicians acting significantly increased during the COVID-19 crisis. Transparent and comprehensive information policy regarding actions and restrictions within the COVID-19 crisis eases patients concerns and improves patients’ confidence in their physicians, which is crucial for a successful treatment’s outcome.

## Introduction

The outbreak of SARS-CoV-2 (“Severe Acute Respiratory Syndrome Coronavirus 2”) and the resulting infectious disease COVID-19 (“Coronavirus Disease 2019”) in Wuhan (Hubei Province, China) began in December 2019. On March 11, 2020, the World Health Organization (WHO) declared COVID-19 an outbreak and emphasized the unprecedented challenge for medical care worldwide [1]. Initiatives to elicit the patient perspective, particularly that of cancer patients, during the COVID-19 outbreak have emerged relatively quickly in 2020. The European Patient Forum is a good example of this as patients use the blog #COVID19 and cancer [2]. To assess the impact of the pandemic on oncological care, a nationwide “flash” survey was conducted among patients with cancer in the Netherlands. The target group was reached electronically, through social media, and website advertisements in the period between 29th March and 18th April 2020. Of the 5302 cancer patients surveyed, 1187 women (22%) had breast cancer and 177 had gynaecological cancer (3%). In this study, about 30% of the patients reported consequences for their gynaecological treatment or follow-up care, especially regarding chemotherapy (30%) and immunotherapy (32%) [3]. Overall, it can be said—also in view of the list of publications on COVID-19 and cancer provided by “the oncologist” [4]—that the patient perspective on the COVID-19 situation has not yet been sufficiently investigated and understood.

Although gynaecology was not primarily involved in the care of COVID-19 patients, the increasing number of infected people has a severe impact on patient care in gynaecology, especially regarding gynaecologic surgery [5]. This development concerns both gynaecological departments in hospitals and gynaecological outpatient clinics.

In this context, the European Federation for Colposcopy (EFC) and the European Society of Gynaecological Oncology (ESGO) stated that an adequate level of care must be provided to patients with lower genital tract pathologies in which therapy cannot be postponed. Furthermore, the resumption of organized vaccination and screening programs must be carefully planned for months to come, ensuring the safety of both patients and healthcare professionals [6].

To minimize the risk of infection within the hospital and outpatient care, the scope of services has been reduced, and extensive measures for infection control and treatment of COVID-19 patients have been established. In addition to that, quarantine, illness periods, and secondments to COVID-19-relevant departments led to a reduction of medical and nursing staff [7]. Thus, at least 28 elective million elective operations were delayed during the first 3 months of the COVID‐19 pandemic worldwide [8],

At the current stage of the pandemic, the impact of these restrictions on the medical care of gynaecological patients has not been studied sufficiently [9, 10]. The aim of this survey was to record the perception of gynaecologic patients of the pandemic, concerns regarding infection or possible inadequate treatment, pandemic safety procedures, and the physician–patient relationship.

## Materials and methods

The Department for Gynecology and Gynecological Oncology of the University Hospital Bonn (UKB) and the Department of Gynecology with Centre for Oncological Surgery Charité Berlin conducted “a validated patient survey” (*n* = 327) for the perception and assessment of gynaecological treatment against the background of the SARS-CoV 2 pandemic (supplement 1). In the period from 1st June to 30th September 2020, 124 questionnaires were completed at the Charité and 203 questionnaires at the UKB or when referred to the UKB by gynaecological outpatient clinics; no patients were excluded. The questionnaires were developed by an interdisciplinary team from the fields of urology, medical ethics, and gynaecology. The completion of the questionnaires was explained and supervised by trained staff. The survey broached the issues of (1) the general perception of the pandemic, (2) concerns about infection during patient care, (3) concerns about inadequate treatment due to pandemic restrictions, (4) perceptions of pandemic-related safety procedures, and (5) the physician–patient relationship, particularly the trust of patients in their treating physicians during COVID-19.

The questionnaires were handed out to patients in the gynaecological outpatient clinics of the University Hospitals in Bonn and Berlin or at the time of referral to the clinic for gynaecology Bonn by the attending gynaecologists. Responses were scored on a Likert scale from one to five. Statistical analysis was performed with StataIC v16.1 software (StataCorp, Lakeway Dr, USA) [11]. For quantitative parameters, the mean SD and range were determined. Significant changes were calculated using the t test and Spearman correlation [12]. *P* values less than 0.05 were considered statistically significant. Data acquisition and analysis were performed in compliance with protocols approved by the ethical committee of the University of Bonn (ethical approval number 443/20). It should be noted that during the period of data collection, the prevalence of COVID-19 in Germany was relatively low compared to the previous months, especially the first wave in March and April 2020 as well as the second wave that started in October 2020 in Germany (peak 18.12.2021: 31,553 new daily cases) [13]. For example, on 01.07.2020, the start of the study, 492 new cases were reported in Germany for that day. In the course of the study, the number of reported cases increased (compare: 30.09.2020: 2445 new daily cases).

## Results

### Patients’ characteristics

The questionnaires were distributed in the outpatient departments of the two clinics in Bonn and Berlin as well as to numerous gynaecologists in private practice in the regions. No distinction was made between the causes of the medical consultation. The patient collective accordingly includes all gynaecological diseases. The questionnaire also does not indicate the type of illness of the participants; only a classification into acute or chronic is requested. No information is available about the planned therapy. Completion of the questionnaires was voluntary for the patients, and there was no personal advantage, which explains the relatively low number of participants for the period. Of the 327 patients surveyed, 99.1% were female, 0.6% male, and 0.3% did not feel represented by the available genders in this questionnaire. Regarding the 0.6% male participants, it should be noted that about 1% of all breast cancers occur in men and that they receive gynaecological care. The median age range was between 41 and 50 years and distributed among the age groups as follows: 18–30 years (14.1%), 31–40 years (20.5%), 41–50 years (21.4%), 51–60 years (20.5%), 61–70 years (12.6%), and > 70 years (10.7%). None of the answers correlated with the age of the patients. 33.0% of patients classified their disease as acute and 26.6% as chronic. 32.1% of patients could not attribute their symptoms to an acute or chronic course. Of the 327 patients, 156 stated to have been tested for coronavirus and 1 patient (0.6%) reported to have tested positive. 6.4% of those tested had not yet known their result (Table 1).

**Table 1:** Patients’ demographics

Characteristics	Value
Patients, n (Bonn, Berlin)	327 (203, 124)
Sex (female, male, divers)	99.1%, 0.6%, 0.3%
Median age of the cohort	41–50
Distribution among the age groups (18–30, 31–40, 41–50, 51–60, 61–70, >70, missing)	14.07%, 20.49%, 21.41%, 20.49%, 12.54%, 10.70%, %0.31
Course of disease (chronic, acute, unknown, missing)	33.03%, 26.61%, 32.11%, 8.26%
Tested on corona (no, yes, missing)	51.99%, 47.7%, 0.31%
If yes (test negative, positive, missing)	92.95%, 0.64%, 6.41%

### General perception of the health situation during the pandemic

The patients clearly differentiated between their general living conditions during the pandemic and the situation during a hospital stay. In everyday life, 41.4% of those surveyed felt insecure about the risk of infection, while 35.9% denied this. 22.7% had no positive or negative opinion on this question. With regard to hospitalization, 30.0% stated that they were generally concerned about a potential infection by COVID-19 during a possible stay in hospital, while 23.0% were neutral, and 47.0% had no concerns about the risk of infection. During a concrete hospital treatment, even 68.1% felt secure, and only 13.5% denied this. While participating in the survey, 20.9% of the participants felt insecure about an infection with COVID-19, and 65.2% felt safe at the time of filling out the questionnaire. Patients who already feared infection to a high degree in their everyday life also shared these fears during a stay in the hospital or medical practice. Overall, the vast majority of patients supported the local clinical decisions to control the pandemic. 74.2% of patients showed understanding of the measures taken, and 63.0% of patients felt that they were part of a solidary and transparent health care system. Only 8.3% denied a feeling of belonging.

### Concerns about pandemic-related deterioration in medical care

The majority of patients have confidence in their treatment and the medical system—also during the COVID-19 pandemic. 64.1% stated that they were not worried about a negative impact on their treatment, nor a loss of quality of care (69.0%), or a poorer prognosis of their disease (65.1%) as a result of the pandemic. A significant proportion of patients, however, believed that their treatment could be affected by the pandemic (18.1%), their treatment options would be limited (16.5%), and their prognosis worsened (16.9%). A statistically significant correlation was found between the uncertainty of patients during hospitalization and their concern about a worsening of the prognosis of their gynaecological disease (*p* < 0.0001).

### Evaluation of hospital internal strategies in the context of COVID-19

The following regulations for patient treatment became necessary to minimize the spread of infections and maintain medical care for all patients in Germany: rescheduling of examinations and planned surgery appointments, reduction of visits by family members to the utmost minimum and the provision of telemedical care for patients as far as possible.

80.0% of the questioned patients showed great understanding towards those regulations. 10.0% of the respondents, however, stated that they had no or only limited understanding of these regulations. The results of the survey show a statistically significant correlation between the level of information and patients' understanding of the above-mentioned regulations established in the context of the COVID-19 pandemic as the rescheduling of appointments (*p* = 0.016) and the prohibition of visits (*p* = 0.0028).

### Doctor–patient relationship

**Fig. 1:**
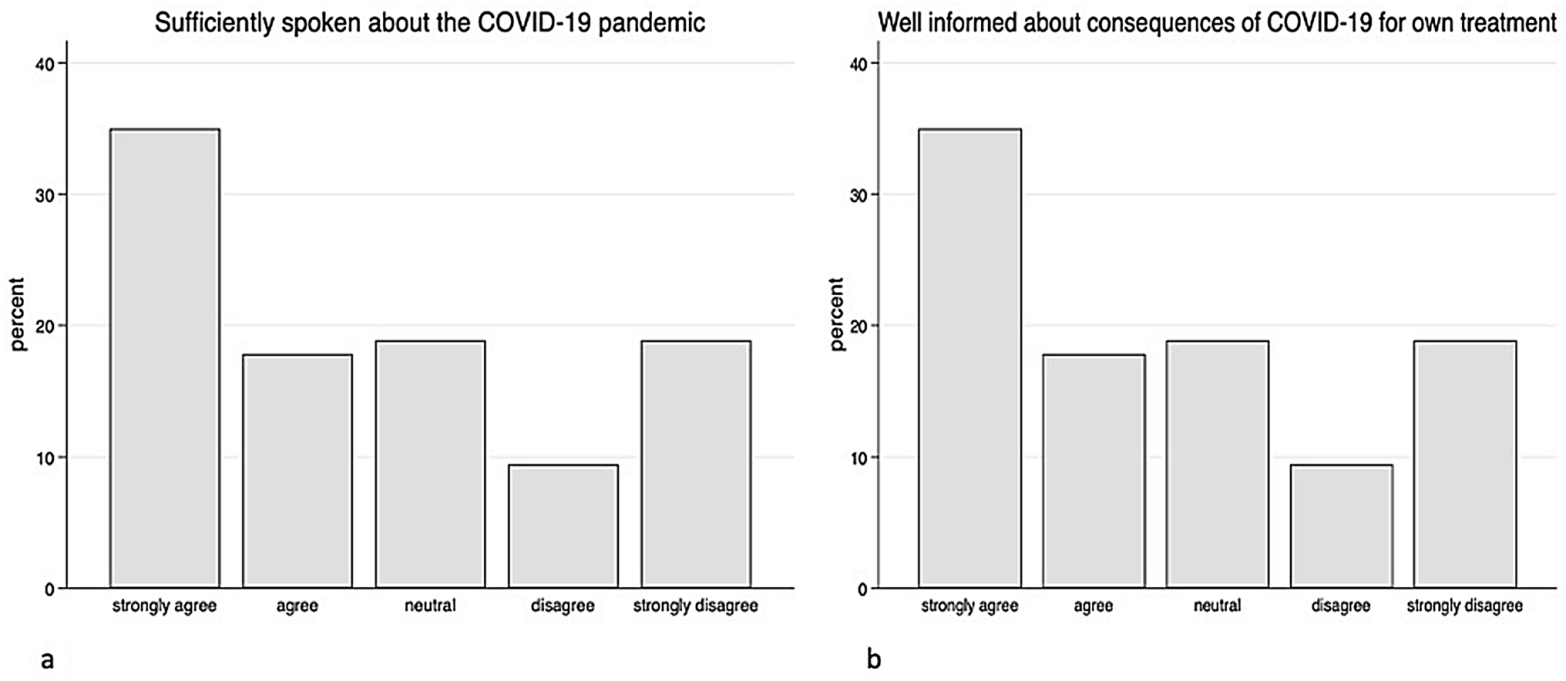
Data showing the patients’ opinion about sufficiently spoken about COVID-19 pandemic (**a**) and how well informed they feel about the consequences of their own treatment (**b**)

**Fig. 2:**
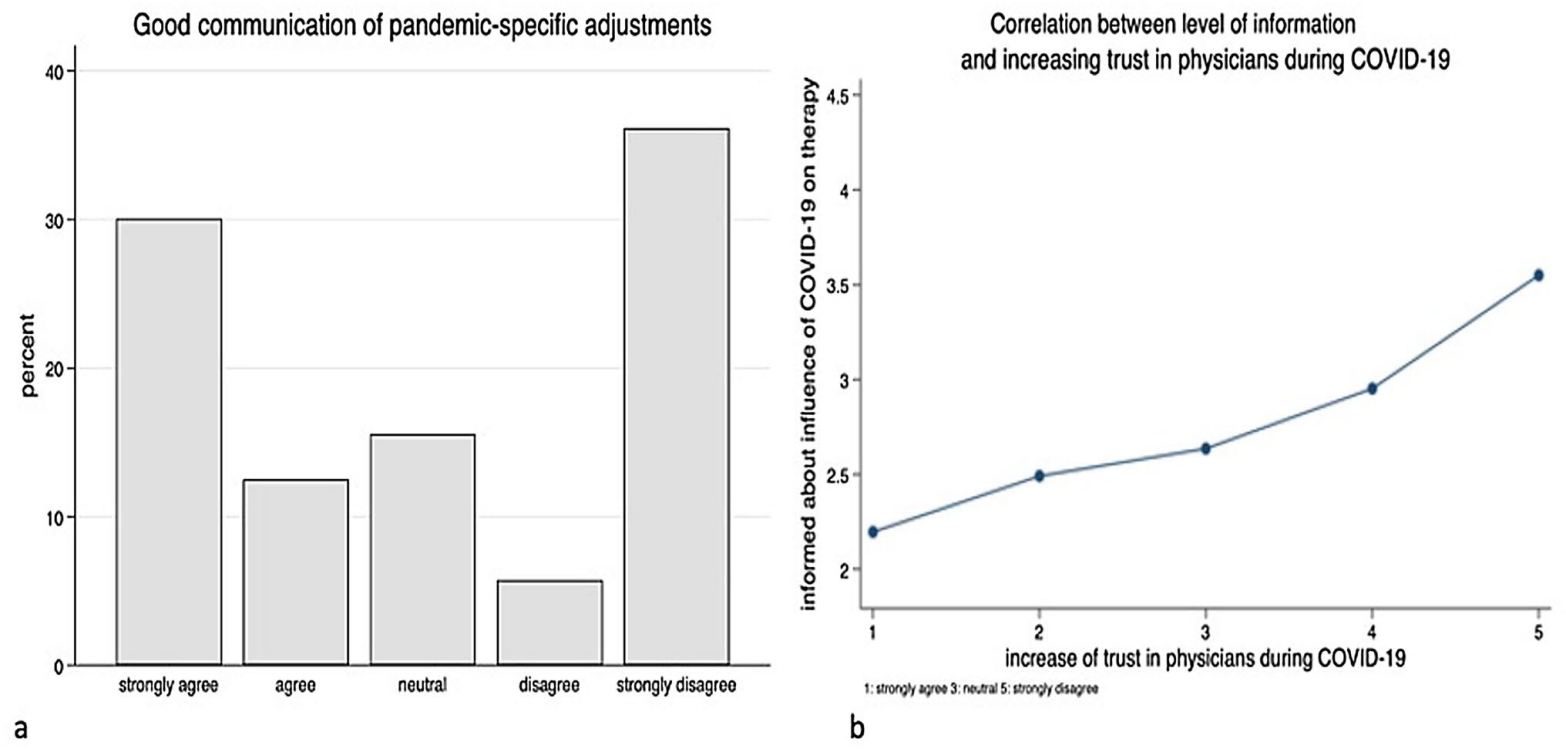
Patient’s perception of the communication concerning pandemic-specific adjustments in the clinics (**a**) and the correlation of the level of information and increasing trust in the gynaecologists during the pandemic (**b**)

**Fig. 3:**
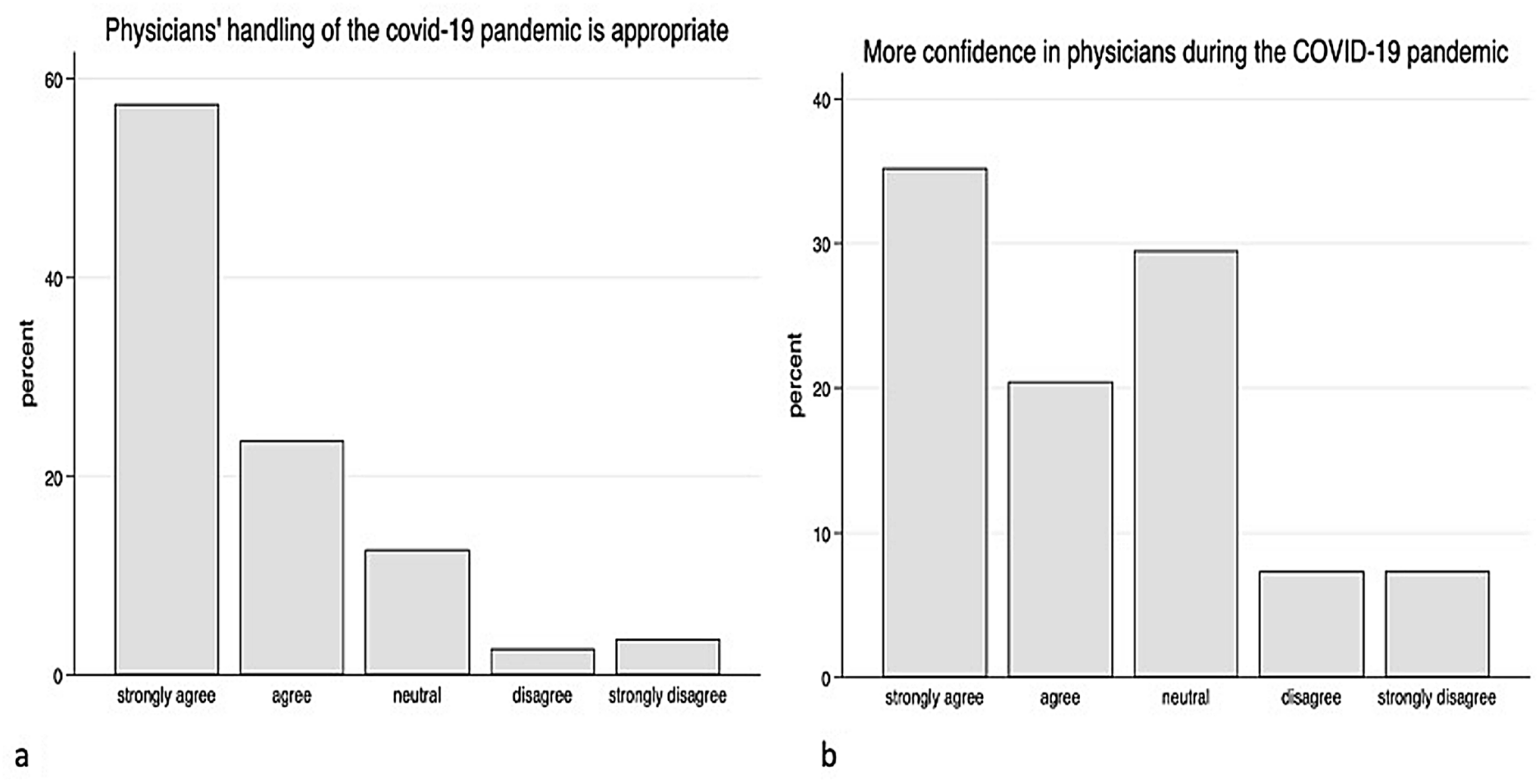
Patients' perception of whether the current clinical measures are good (**a**) and assessment of this based on their confidence in the doctors (**b**)

The survey showed that patients perceived the flow of information in this pandemic situation very differently. While 52.9% of the patients have the impression that the situation has generally been sufficiently discussed, 28.3% stated that this was rather not the case. 18.8% of the patients did not make a clear positive or negative statement (Fig. 1a). The patients’ perception of changes in their own treatment was comparable: 54.0% of the patients felt well informed about the consequences of their own treatment, 30.8% felt that the information was insufficient, and 15.2% did not formulate a clear statement about it (Fig. 1b). With regard to the communication of pandemic-specific adaptations of work processes in medical facilities such as university clinics, a larger deviation can be observed. 42.6% of the patients felt well informed; however, 41.9% stated that they had not received any information about it, and 15.5% were neutral towards this question (Fig. 2a). The survey clearly shows a statistically significant positive correlation between the patient trust in their doctors and their level of information (Fig. 2b). As a result, 81.1% of the patients consider the way doctors react with the current situation to be appropriate, only 6.3% disagreed, and 12.6% of respondents had no positive or negative opinion on this question (Fig. 3a). In addition, 55.7% of the patients stated that they trusted their physicians even more during the pandemic situation than before (Fig. 3b).

## Discussion

This is the first study investigating the perception of gynaecologic patients during the COVID-19 pandemic in two gynaecological departments and outpatient clinics in Germany. It shows that a comprehensive information policy is crucial to maintain a trustful relationship with the hospital. Furthermore, the strength of this study is that it is the first study to present the patients' concern of the risk of infection during the hospital stay. The impact of the current pandemic on the healthcare system is currently the subject of a broad public and scientific debate. On March 13, 2020, German hospitals were politically called upon to ensure that from March 16, 2020, all plannable admissions, operations, and interventions are postponed indefinitely if this is medically justifiable. The hospitals should primarily focus on the care of current and future COVID-19 patients [14] and provide intensive care. At the same time, public life in Germany became severely restricted. Since the end of April, this state of affairs has gradually reversed, and in early May, some of the clinics returned to a controlled mode. We found a widely varying degree of insecurity in everyday life and during hospitalization. Remarkably, the general level of concern about COVID-19 infection is higher in everyday life than during a hospital stay. On one hand, this difference could be explained by the fact that patients in the hospital must state beforehand that they do not have symptoms typical of COVID-19 and are, at least before hospitalization, tested for the virus. In addition, it can be assumed that in the hospital, the concern about their own symptoms outweighs the concern of an infection with COVID-19. Furthermore, the most medical consultations have to be made, and the risk of a possible infection is, therefore, more likely to be tolerated than in a normal everyday situation, which is more likely to be avoided. As mentioned in the results, only one patient tested positive for coronavirus during the hospital stay. The low rate of positively tested patients results from the time period of the survey. In addition, all patients were asymptomatic with regard to COVID-19 symptoms at the time of the clinic stay.

It can be assumed that many of these patients appeared with first-time symptoms. Of the 327 patients, 156 stated to have been tested for coronavirus and 1 patient (0.6%) reported to have tested positive. 6.4% of those tested had not yet known their result (Table 1). The low rate of positively tested patients results from the time period of the survey. In addition, all patients were asymptomatic with regard to COVID-19 symptoms at the time of the clinic stay.

The majority of respondents accept telemedicine and e-health services for outpatient treatment of a COVID-19 infection regardless of age [15]. In addition, such technologies could also be used for the initial diagnosis of potentially infected individuals, for medical care of mild forms of COVID-19 cases, and for medical care of people in quarantine or in remote areas [16]. These services, however, are far from being available to all patients and face new ethical challenges. For example, physicians would need to prepare well in advance of video consultation for difficult situations, such as the virtual transmission of an unfavourable diagnosis [17]. The pandemic is an opportunity to develop the necessary technologies and infrastructure to improve telemedicine [18]. In our cohort, the evaluation of telemedical approaches showed 54.6% of patients able to accept such medical services, but 23.4% rejected this form of patient care.

The study situation regarding the quality of gynaecological treatment during the COVID-19 pandemic is still very poor. Invasive, high-maintenance treatment methods, in particular, are viewed critically, as the fair and necessary distribution of available resources is at stake against the background of increasingly limited medical resources [11].

The study situation regarding the quality of gynaecological treatment during the COVID-19 pandemic is still very poor. In particular, the implementation of invasive and care-intensive treatment methods must be critically considered, given the currently limited resources [19].

The significant impact of the pandemic on both physicians and patients cannot be denied, and COVID‐19 has dramatically affected the care of patients with gynaecological cancer. The extent of these effects is related to the burden of COVID‐19, particularly the availability of local resources [20]. A study in the Netherlands reported that cancer diagnoses have decreased dramatically during the pandemic, suggesting that patients cannot reach hospitals and are diagnosed at advanced stages [21]. Compared to these results, our study shows a high level of contentment with gynaecological care within the cohort of our patients.

The respondents generally consider the handling of the COVID-19 pandemic to be appropriate (74.2%). Only 6.8% of the patients rejected the current regulations. In particular, the behaviour of physicians is positively emphasized. Confidence in physicians has even increased in this special situation. The most important element here is comprehensive information for patients [22]. This concerns the enlightenment about necessary regulations in the hospital organization, as well as possible changes and possible effects in the individual therapy. A study by Nelson et al. [23] provided convincing evidence of an increase in anxiety and depression symptoms compared to historical normative data, indicating a clinically significant increase in societal mental health problems during the COVID-19 pandemic. Therefore, a trustful relationship between physician and patient seems to be even more important during the COVID-19 pandemic. This may be accomplished by absolute transparency and information about all regulations in the hospital and during therapy. Another study demonstrated that the confusion regarding information about COVID-19 was significantly higher among those who had lower health literacy [24]. Therefore, we think that our practice of transparency led to increased confidence in the decisions of the medical staff, as well as in the entire health care system. The majority of our patients surveyed had no concerns that the implementation and outcome of their treatment could be affected by the COVID-19 pandemic. These concerns could certainly be further addressed by improving comprehensive education about the impact of the pandemic on individual health care. It is the responsibility of treating physicians to provide comprehensive information on the consequences of the COVID-19 pandemic, in addition to the prescribed education. This is confirmed by the broad consensus of measures, such as postponing appointments or prohibiting visits by patients’ relatives. Remarkably, our data show that the trust in the German health care system has not decreased during the COVID-19 pandemic, which is exceptional as trust generally deteriorates in situations of high uncertainty and ambiguous behaviour [10, 25]. Our findings are consistent with the findings of a recent study showing that public trust in the health care system is positively related to differences in compliance behaviour during a pandemic crisis situation [26].

Overall, it can be concluded that there is a high level of confidence in the healthcare system in Germany, even in the current exceptional situation. Nevertheless, better communication strategies with patients and the initiation of patient materials, such as brochures, may improve patients’ understanding to overcome the uncertainness of the management of gynaecological diseases.

## Data Availability

The applied questionnaire is attached.
